# Contact Angle and Cell Adhesion of Micro/Nano-Structured Poly(lactic-*co*-glycolic acid) Membranes for Dental Regenerative Therapy

**DOI:** 10.3390/dj9110124

**Published:** 2021-10-20

**Authors:** Naoyuki Kaga, Hiroki Fujimoto, Sho Morita, Yuichiro Yamaguchi, Takashi Matsuura

**Affiliations:** 1 Section of Fixed Prosthodontics, Department of Oral Rehabilitation, Fukuoka Dental College, Fukuoka 814-0193, Japan; fujimotoh@college.fdcnet.ac.jp (H.F.); moritas@college.fdcnet.ac.jp (S.M.); youdesu@college.fdcnet.ac.jp (Y.Y.); matsuurt@college.fdcnet.ac.jp (T.M.); 2 Oral Medicine Research Center, Fukuoka Dental College, Fukuoka 814-0193, Japan

**Keywords:** micro/nano pattern, poly(lactic-*co*-glycolic acid), contact angle, cell adhesion, nanoimprint, guided tissue regeneration, guided bone regeneration

## Abstract

Biodegradable membranes are used in regenerative dentistry for guided tissue regeneration (GTR) and guided bone regeneration (GBR). In this study, patterned poly(lactic-*co*-glycolic acid) (PLGA) membranes with groove, pillar, and hole structures were successfully fabricated by thermal nanoimprinting. Their surfaces were evaluated for topography by scanning electron microscopy and laser microscopy, for hydrophobicity/hydrophilicity by contact angle analysis, and for MC3T3-E1 cell adhesion. The sizes of the patterns on the surfaces of the membranes were 0.5, 1.0, and 2.0 μm, respectively, with the height/depth being 1.0 μm. The pillared and holed PLGA membranes were significantly more hydrophobic than the non-patterned PLGA membranes (*p* < 0.05). However, the 0.5 μm- and 1.0 μm-grooved PLGA membranes were significantly more hydrophilic than the non-patterned PLGA membranes (*p* < 0.05). The 0.5 μm-grooved, pillared, and holed membranes exhibited significantly superior adhesion to the MC3T3-E1 cells than the non-patterned PLGA (*p* < 0.05). These results suggest that patterned PLGA membranes can be clinically used for GTR and GBR in the dental regeneration field.

## 1. Introduction

In regenerative dentistry, the emergence of restoration of alveolar bone lost due to periodontal disease and dental implants is increasing the demand for bone regeneration to combat bone defects. The membranes used in guided tissue regeneration (GTR) and guided bone regeneration (GBR) are either biodegradable or non-absorbable depending upon their material of construction [1,2]. In the case of the latter, a secondary surgery is required for removal, and owing to problems such as damage to regenerated tissue and risk of infection, the use of absorbent membranes has become mainstream in recent years [3].

Currently, biodegradable materials such as polylactic acid (PLA), polyglycolic acid (PGA), polycaprolactone (PCL), and poly(lactic-*co*-glycolic acid) (PLGA) are used as scaffolding materials for tissue reconstruction [4,5,6,7]. Additionally, biodegradable membranes based on collagen have been clinically applied [1,8]. Animal-derived collagen membranes exhibit high biocompatibility and biodegradability and excellent cell affinity. However, they possess low mechanical strength, and it is difficult to control the rate of their decomposition in the living body [9].

Research has also been conducted on the behavior of cells against biodegradable polymer-based GTR and GBR membranes. It has been reported that when osteoblasts were cultured on a PLGA membrane, cell elongation was suppressed on a flat surface, whereas it was promoted on a sparse and porous surface [10]. Further, when fibroblasts were cultured on a poly L-lactic acid (PLLA) membrane, cell proliferation was promoted on sparse surfaces and suppressed on dense surfaces [11]. In addition, culturing epithelial cells on PLA/PLGA membranes with a dense surface structure suppresses cell invasion into the membrane [12]. However, the surface structure of these bioabsorbable polymer membranes is porous. The micro/nanosized grain structure controls the cell proliferation rate, and the nanosized line and groove structure controls the cell migration, division, and growth orientation [13].

In case a micro/nanopattern structure could be imparted to the membrane composed of PLGA (which is a bioabsorbable polymer), it could be absorbed in vivo and enhance the functions of cell adhesion, elongation, proliferation, and differentiation in the membrane. In this study, we prepared membranes with micro/nanopattern structures using PLGA by following the method reported in our previous works [14,15] and evaluated their surface characteristics, including surface shape, contact angle, and cell adhesion.

## 2. Materials and Methods

### 2.1. Fabrication of the Micro/Nano-Structured PLGA Membrane

**Figure 1:**
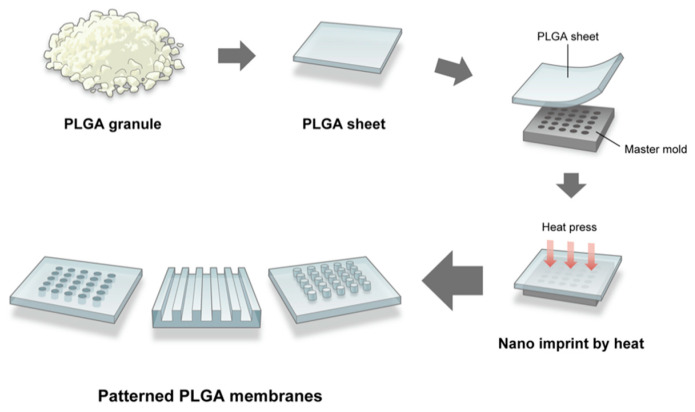
Schematic illustrating the fabrication process of the micro/nanopatterned PLGA membranes.

Figure 1 schematically illustrates the fabrication process of the micro/nanopatterned PLGA membranes. Each of nine PLGA membrane sheets (5 × 5 mm^2^) with grooves (ridge widths: 0.5, 1, and 2 μm; depth: 1 μm), pillars (diameters: 0.5, 1, and 2 μm; height: 1 μm), and holes (diameters: 0.5, 1, and 2 μm; depth 1 μm) on their surfaces were fabricated using a quartz trial mold (master mold; Kyodo International Inc., Kawasaki, Japan). PLGA granules (BMG Inc., Kyoto, Japan) were processed into PLGA sheets using a thermal nanoimprinter (HC300-01, AS ONE Inc., Osaka, Japan) at 130 °C and 2 MPa. Thereafter, the PLGA sheet was set on the trial mold and pressed at 2 MPa and 85 °C for 5 min using the aforementioned heat press and gradually cooled to 23 °C.

### 2.2. Scanning Electron and Laser Microscopy

The micro/nano-structured PLGA membranes were sputter-coated with Au-Pt and observed via scanning electron microscopy (SEM) using a JSM-6330F electron microscope (JEOL, Tokyo, Japan) operated at an accelerating voltage of 5 kV. The surface topography of the Au-Pt-coated micro/nano-structured PLGA membranes was observed using a 3D laser microscope (VK-X200; Keyence, Osaka, Japan).

### 2.3. Contact Angle of the Micro/Nano-Structured PLGA Membranes

The hydrophilicity of each sample surface was investigated using a B100 contact angle meter (ASUMI GIKEN Limited, Tokyo, Japan). Droplets of ultrapure water (2 μL) placed on each sample were photographed horizontally, and the contact angles were measured. Measurements were performed five times on each sample at a temperature and humidity of 24 °C and 57%, respectively.

### 2.4. Cell Culture

MC3T3-E1 cells were provided by the RIKEN BRC through the National BioResource Project, in conjunction with MEXT, JAPAN (Accession Numbers: R21-0386). MC3T3-E1 (RCB1126) cells were cultured in MEMalfa with L-glutamine and phenol red (MEMα; FUJIFILM Wako Pure Chemical Corp., Tokyo, Japan) containing 10% fetal bovine serum (FBS; CELLect GOLD, Australia; MP Biomedicals Inc., Solon, OH, USA) and a 1% penicillin/streptomycin/amphotericin B suspension (Wako Pure Chemical Industries, Ltd., Osaka, Japan) as an antibiotic-antimycotic solution. The cell culture was maintained in a humidified incubator at 37 °C with 5% CO_2_ and 95% air. The cell growth was observed daily, and the medium was replaced every 3 d for uninhibited growth. At 70% cell confluence, the cells were treated with a cell detachment reagent (Accumax; Funakoshi Co., Ltd., Tokyo, Japan). Single floating cells were counted using a hemocytometer for subsequent assays.

### 2.5. Cell Adhesion Test

Cell adhesion tests were performed to estimate the kinetics of cell adhesion on the patterned PLGA membranes. The patterned PLGA membranes were immersed in the culture medium. Thereafter, MC3T3-E1 cells were seeded on the membranes at a density of 5000 cells/cm^2^ and incubated at 37 °C in a humidified atmosphere of 5% CO_2_ and 95% air for 3 h to facilitate cell adhesion. Thereafter, the cells were fixed using 2.5% glutaraldehyde. Subsequently, cell adhesion was observed by an optical microscope (KX41; Olympus Corp., Tokyo, Japan) connected to a digital camera. The cells that had attached onto the membranes following incubation for 3 h were evaluated from photographs of each grooved, pillared, and holed surface using ImageJ. The cell density on each patterned field (5 × 5 mm^2^/field) was calculated. The samples were stained using Mayer’s Hematoxylin solution (Wako Pure Chemical Industries, Ltd., Osaka, Japan) following incubation for 3 h for morphological observations, which involved the use of a light microscope (KX41) and a Lumix DC-G 100 camera to capture the images.

### 2.6. Confocal Laser Scanning Microscopy

The actin filaments of MC3T3-E1 cells were observed using a confocal laser scanning microscope (CLSM) 3 h after seeding on the samples. The cells were washed twice in phosphate-buffered saline (PBS) and fixed for 10 min in 4.0% formaldehyde in PBS. The cells were permeabilized in 0.1% Triton X-100 (Sigma-Aldrich, Tokyo, Japan) in PBS for 5 min and washed three times using PBS. Blocking was performed in PBS containing 5% bovine serum albumin for 30 min, and the cells were washed once using PBS. Subsequently, the cells were fluorescently stained for detection of F-actin at 37 °C for 30 min using Alexa Fluor 488-phalloidin (Thermo Fisher Scientific, Tokyo, Japan). The samples were observed using an ECLIPSE Ti CLSM (Nikon Corp., Tokyo, Japan).

### 2.7. Statistical Analysis

Statistical data analysis was performed using GraphPad Prism version 8.1.2 (GraphPad Software, Inc., La Jolla, CA, USA). All data are reported as means and standard deviations. One-way ANOVA and Dunnett’s multiple comparison tests were used to identify statistical differences. Differences were considered statistically significant at *p* < 0.05.

## 3. Results

### 3.1. SEM Images of the Patterned PLGA Membranes

**Figure 2:**
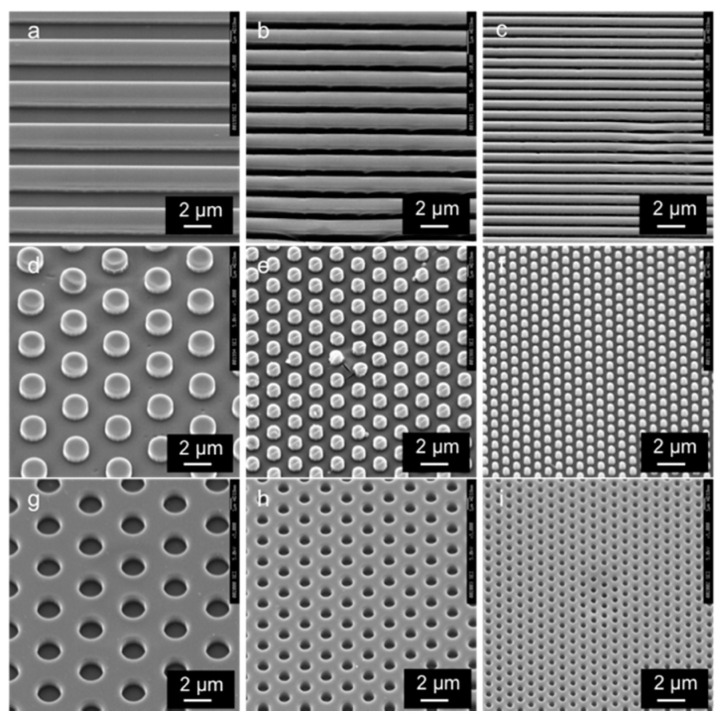
SEM images of the patterned PLGA membranes. Groove widths of (**a**) 2.0, (**b**) 1.0, and (**c**) 0.5 μm; pillared widths of (**d**) 2.0, (**e**) 1.0, and (**f**) 0.5 μm; and hole widths of (**g**) 2.0, (**h**) 1.0, and (**i**) 0.5 μm. Scale bar = 2.0 μm.

Figure 2 shows the SEM images of the micro/nano-structured PLGA membranes. Grooves, pillars, and holes were transcribed from the corresponding fine shape onto the membranes, and the surface of each pattern was uniformly smoothed at the micro/nano scale.

### 3.2. Laser Microscope Images of the Patterned PLGA Membranes

**Figure 3:**
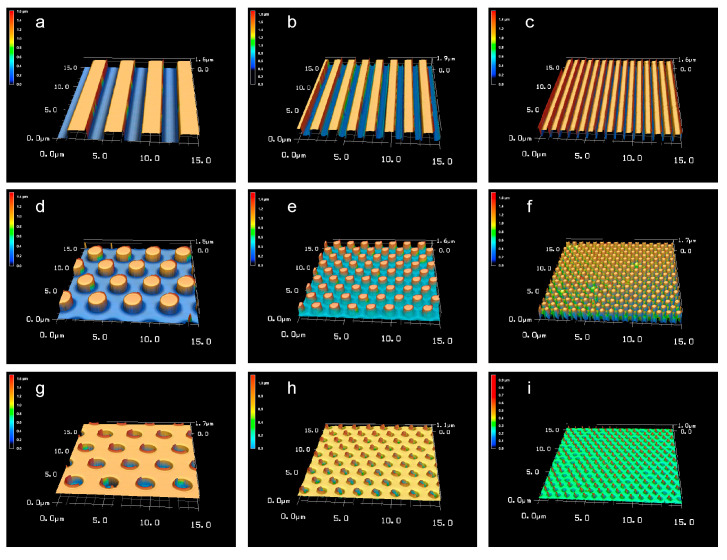
Laser microscope images of the patterned PLGA membranes. Groove widths of (**a**) 2.0, (**b**) 1.0, and (**c**) 0.5 μm; pillar widths of (**d**) 2.0, (**e**) 1.0, and (**f**) 0.5 μm; and hole widths of (**g**) 2.0, (**h**) 1.0, and (**i**) 0.5 μm. Scale bar = 2.0 μm.

Figure 3 and Table 1 show the laser microscope images and pattern sizes of the micro/nano-structured PLGA membranes. The groove widths were 2.0 ± 0.01, 1.0 ± 0.01, and 0.5 ± 0.02 μm. The pillar widths were 2.0 ± 0.01, 1.0 ± 0.03, and 0.5 ± 0.04 μm. The hole widths were 2.0 ± 0.03, 1.0 ± 0.04, and 0.5 ± 0.05 μm. The height or depth of each pattern was approximately 1.0 μm.

### 3.3. Measurement of Contact Angles of the Patterned PLGA Membranes

**Figure 4:**
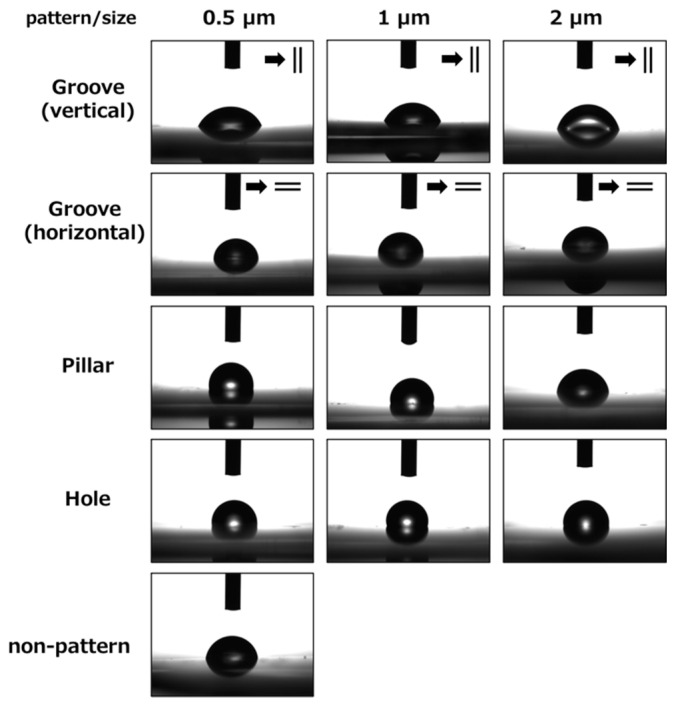
Contact angle images for the micro/nanopatterned PLGA membranes.

**Figure 5:**
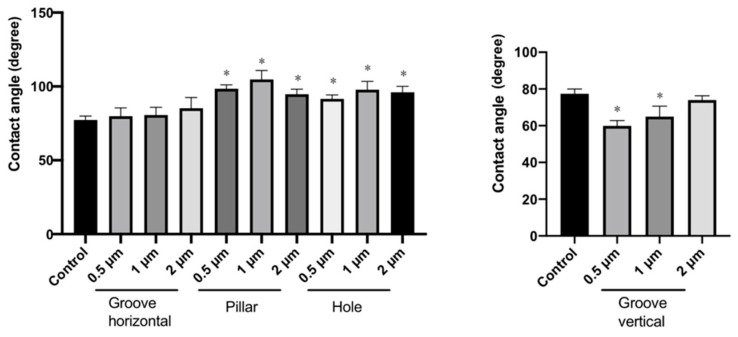
Contact angle measurement data. The contact angle was normalized to that of the control (non-pattern). (* indicates *p* < 0.05).

Figure 4 and Figure 5 show the contact angle images and data of the patterned PLGA membranes, respectively. The contact angle for the grooved PLGA membranes (horizontal view for groove) was not significantly different from that of the non-patterned PLGA membrane. However, the 0.5 μm- and 1.0 μm-grooved PLGA membranes (vertical view for groove) were significantly more hydrophilic than the non-patterned PLGA membrane (*p* < 0.05). The mean contact angles of the 0.5 μm-, 1 μm-, and 2 μm-pillared membranes were 98.47°, 104.78°, and 95.86°, respectively. The mean contact angles of the 0.5 μm-, 1 μm-, and 2 μm-holed membranes were 91.58°, 97.82°, and 99.42°, respectively. The mean contact angle of the non-patterned membranes was 77.34°. The pillared and holed PLGA membranes were significantly more hydrophobic than the non-patterned PLGA membrane (*p* < 0.05). However, the contact angles were not significantly different among the pillared and holed patterns.

### 3.4. Cell Adhesion test on the Patterned PLGA Membranes

**Figure 6:**
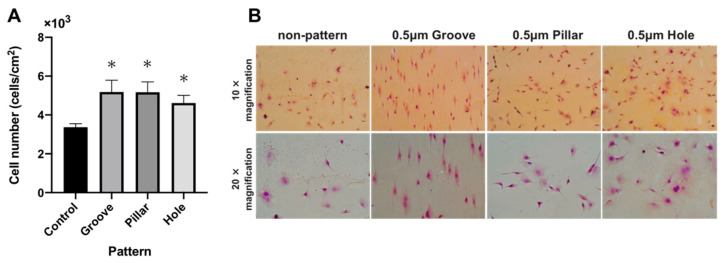
(**A**): The number of MC3T3-E1 cells attached onto the non-patterned (control) and patterned PLGA membranes following 3 h of incubation. (**B**): Light micrographs of MC3T3-E1 cells on non-patterned or patterned PLGA membranes following 3 h of incubation.

Figure 6 shows the number of the MC3T3-E1 cells attached to the non-patterned (control) and patterned PLGA membranes following incubation for 3 h. The number of cells attached to the grooved, pillared, and holed PLGA membranes was significantly higher than that for non-patterned PLGA membranes (*p* < 0.05). Numerous adherent cells were extended along the pattern (Figure 6B).

### 3.5. CLSM Images of Actin Filaments

**Figure 7:**
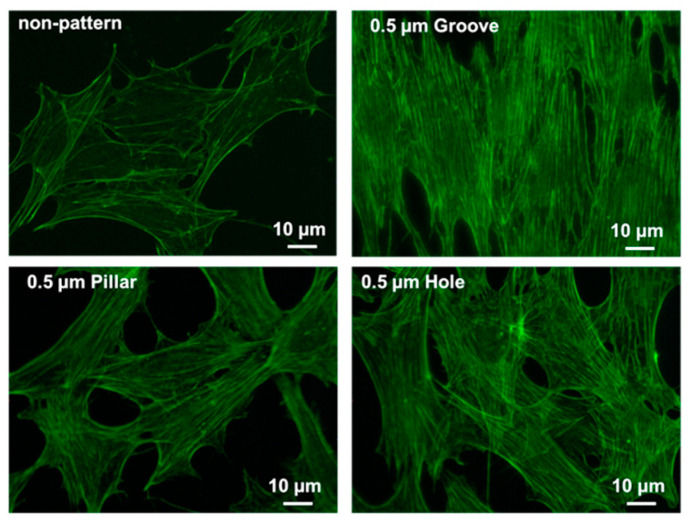
CLSM images of actin filaments of MC3T3-e1 cells on non-patterned or patterned PLGA membranes.

Figure 7 shows the CLSM images of actin filaments of MC3T3-E1 cells on non-patterned and patterned PLGA membranes following 3 h of incubation. On the grooved PLGA membrane, numerous actin filaments were observed to be oriented along the groove. On the pillared and holed PLGA membranes, actin filaments were observed to extend radially. On the non-patterned PLGA membrane, actin filaments were observed to be thin, short fibers. Larger number of actin filaments had spread out in a fibrous form on the patterned PLGA membranes than on the non-patterned PLGA membrane. The morphology was influenced by the pattern.

## 4. Discussion

In the present in vitro study, we successfully prepared PLGA membranes with grooved, pillared, and holed structures by thermal nanoimprinting. The patterns created on the PLGA membranes influenced the cell adhesion and altered the cytoskeleton. The patterns crafted on the surfaces of the membranes were of three sizes: 0.5, 1, and 2.0 μm, with the height/depth remaining constant at 1.0 μm. We observed that the smaller the size of the pattern, the more severe the reproducibility of the width and height. However, it has been suggested that a diverse set of pattern structures can be imprinted onto the surface of dental biomaterials by adjusting the pressure, temperature, and pressing time [14,15]. Studies have reported the imprinting of the surface structures of cell scaffolds of bioabsorbable polymers. The growth and differentiation of C2C12 myoblasts were enhanced by grooved PLGA films (ridge/groove: 0.8 μm; depth: 0.6 μm) compared to the untreated PLGA [16]. Furthermore, two times as many human bronchioalveolar carcinoma cells adhered to a photo-decomposable polymer film with a holed structure than to the same scaffold lacking the holed structure [17]. Nano-pillars or holes have been reported to improve water repellency. Shiozawa et al. reported that titanium substrates with micro- or nano-sized surface topography have lower hydrophilicity, but higher cell proliferation ability [13].

In our study, patterned PLGA membranes were fabricated by nanoimprinting. The heating process may influence the degradation times and mechanical characteristics of PLGA membranes. The resorption times of resorbable membranes were approximately 4–8 weeks [18]. Nadja et al. reported that the membranes were inserted in the backs of rats and the degradation, visibility, and tissue integration of the membranes were investigated [19]. PLGA membranes were mostly visible and exhibited insignificant degradation at 4 weeks. PLGA membranes exhibited complete integration and tissue ingrowth by 26 weeks during the histological analysis. We did not investigate the decomposition or mechanical properties. Therefore, a histological investigation will be required to determine whether the pattern influences the decomposition or mechanical properties following thermal nanoimprinting.

In this study, the PLGA membranes with pillar- and hole-structures were found to be more hydrophobic than the non-patterned PLGA membrane. However, a significantly larger number of the MC3T3-E1 cells adhered to the 0.5 μm-sized pillared and holed patterned PLGA membranes than to their non-patterned counterpart (Figure 6A).

The role of the GTR or GBR membranes is to perform block epithelial tissue invasion and promote cell proliferation and differentiation at the tissue regeneration site. The material itself does not exhibit the control functions of cell adhesion, alignment, proliferation, or differentiation. A membrane exhibiting such functions would be novel. Owen et al. reported that although epithelial cells proliferated on PLGA with sandblasted-acid-etched topographies, the number of cells did not significantly increase over 5 d, whereas osteoblasts proliferated and were aligned on PLGA with a grooved structure [20]. Poly (d, l-lactic acid) (PDLLA) and PLGA composite membranes can function as a physical barrier that inhibits cell migration and proliferation in vivo [21]. Therefore, it was hypothesized that because of thermal nanoimprinting, the micro-nanopattern formed on the PLGA membrane may physically impede aspects of cell infiltration and function such as cell adhesion, proliferation, and alignment.

The bioabsorbable polymers PLA, PGA, and PLGA are widely applied in the field of regenerative medicine. These materials temporarily assist until the tissue damage is healed, whereafter they are quickly decomposed and absorbed. In addition, it is desirable that the cells function as a scaffold for biological tissues for adhesion and proliferation [22,23]. Therefore, we observed the morphology of the adherent cells and the formation of actin filaments by LM (Figure 6B) and CLSM (Figure 7). On the grooved PLGA membrane, cells were observed to be oriented along the groove patterns. Numerous cytoskeletons were formed, and the cell alignment was observed. On pillared and holed PLGA membranes, the cells were expanded, and the formation of larger numbers of fine actin filaments compared to the non-patterned PLGA membrane was confirmed. Actin stress fibers bind to cell membranes through interaction with the focal adhesions of integrin. This is a complex adhesion mechanism that proceeds via adhesive proteins such as talin and vinculin [24,25]. Consequently, further investigations are required to ascertain the mechanism of adhesion between cells and patterned structures in detail [26].

Titanium membranes have been widely used clinically as non-absorbable membranes for GTR and GBR [27,28]. Recently, hole structures (diameter: 20 μm) imprinted onto a titanium membrane were clinically applied [29,30]. Depending on the microstructure, the most immature fibroblasts proliferated in the scaffold through the perforated hole using the imparted microstructure as a scaffold [30]. However, non-absorbable membranes require secondary surgery for removal and carry the risk of postoperative pain and infection [31]; in addition, the patient’s surgical invasion is high. By imparting a two-layer structure onto PLGA membranes with different scaffoldings for the osteoblasts on the bone defect side and epithelial cells on the gingival side, cell proliferation can be controlled [7]. The possibility of printing different patterns on the bone defect side and the epithelial side of the membrane may further assist regenerative therapy.

**Figure 8:**
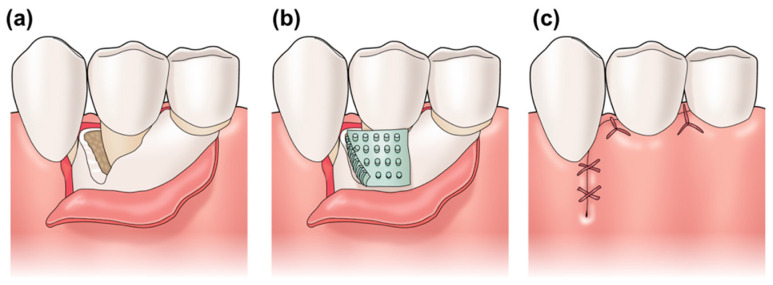
Diagram for clinical application of the patterned PLGA membrane. (**a**) Bone defect caused by periodontal disease. (**b**) Using the micro/nanopatterned PLGA membrane in GTR. (**c**) Suture was performed.

The physical or chemical surface properties of biomaterials are known to selectively affect cell adhesion, proliferation, and differentiation. In particular, cell adhesion is considered to influence cell differentiation. Furthermore, cell adhesion to biomaterials strongly depends on the nanoscale topography and chemical structure of the material surface [32]. Previous studies have reported successful micro-nanopatterning of the surfaces of materials such as titanium [33], calcium phosphate [34], and gelatin [35]. Applying the advancements made in this study will make it possible to fabricate scaffolds with micro/nanopatterned structures that promote osteoblast migration and periodontal tissue regeneration on the bone defect side. On the epithelial side, they may suppress early adhesion or downgrowth of epithelial cells [36] by providing micro/nanopatterns that inhibit the migration of epithelial cells. We believe that patterned PLGA membranes can be clinically applied in dental regenerative therapy, particularly in GTR and GBR (Figure 8). Applying the advancements made in this study, cell migration may be controlled on the epithelial side and the bone defect side. Furthermore, it may assist periodontal tissue regeneration to a significant extent.

The present study has some limitations. It has demonstrated that micro/nano patterning the surface structure of PLGA influences osteoblast adhesion. For actual clinical use, long-term in vivo compatibility of absorbable membranes of GTR and GBR is essential. In addition, it is essential to perform detailed material evaluations of PLGA membranes fabricated by our method. Transferring patterns to biomaterials using our method is simple and inexpensive. However, the drawback of the method is that the membranes have the dimensions of the master mold. The larger the size of the master mold, the more difficult it is to transfer.

## 5. Conclusions

We prepared groove-, pillar-, and hole-patterned PLGA membranes by thermal nanoimprinting. Their surfaces were evaluated for topography by scanning electron microscopy and laser microscopy, for hydrophobicity/hydrophilicity by contact angle analysis, and for MC3T3-E1 cell adhesion. The contact angle of the pillar-and hole-patterned PLGA membranes was higher, indicating increased hydrophobicity. However, the number of MC3T3-E1 cells that adhered to the grooves, pillars, and holes of the patterned PLGA membranes was significantly higher than that for the non-patterned PLGA membrane. Through the method discussed in this paper, the functions of cell adhesion, alignment, proliferation, and differentiation may be imparted to the bioabsorbable membrane. Consequently, cell migration may be controlled on the epithelial and bone defect sides. In the future, the potential of proliferation and differentiation of osteoblasts should be investigated through the response to epithelial cells.

## Figures and Tables

**Table 1:** Pattern sizes of the micro/nano-structured PLGA membranes (Figure 3a–i).

Pattern Type		Groove			Pillar			Hole		
		a	b	c	d	e	f	g	h	i
Pattern size	Width (μm)	2.0 ± 0.01	1.0 ± 0.01	0.5 ± 0.02	2.0 ± 0.01	1.0 ± 0.03	0.5 ± 0.04	2.0 ± 0.03	1.0 ± 0.04	0.5 ± 0.05
	Height (μm)	1.0 ± 0.01	1.0 ± 0.01	1.0 ± 0.03	1.0 ± 0.02	1.0 ± 0.06	0.9 ± 0.05	1.0 ± 0.07	0.9 ± 0.05	0.9 ± 0.7

## Data Availability

Not applicable.
